# Effect of sodium hypochlorite, ethylenediaminetetraacetic acid, and dual-rinse irrigation on dentin adhesion using an etch-and-rinse or self-etch approach

**DOI:** 10.1038/s41598-024-57009-x

**Published:** 2024-03-15

**Authors:** Matej Par, Tobias Steffen, Selinay Dogan, Noah Walser, Tobias T. Tauböck

**Affiliations:** 1 Department of Endodontics and Restorative Dentistry, School of Dental Medicine, University of Zagreb, Gunduliceva 5, 10000 Zagreb, Croatia; 2 Private Dental Practice, Zurich, Switzerland; 3 Department of Conservative and Preventive Dentistry, Center for Dental Medicine, University of Zurich, 8032 Zurich, Switzerland

**Keywords:** Endodontic irrigations, NaOCl, EDTA, Dual-rinse, Micro-tensile bond strength, Dentin adhesion, Failure analysis, Biomaterials, Biomedical materials

## Abstract

The aim was to investigate the influence of endodontic irrigation solutions and protocols on the micro-tensile bond strength (μTBS) to dentin using an etch-and-rinse (ER) or self-etch (SE) adhesive approach. Eighty extracted human molars were ground to dentin. After pretreating for 27 min (21 min–3 min–3 min) with five different endodontic irrigation protocols (Group 1: NaOCl–EDTA–NaOCl; Group 2: NaOCl–NaOCl–EDTA; Group 3: NaOCl–NaCl–NaOCl; Group 4: Dual Rinse–Dual Rinse–Dual Rinse; Group 5: NaCl–NaCl–NaCl), an ER (Optibond FL, Kerr) or a SE (Clearfil SE Bond, Kuraray) adhesive system was applied. After light-curing, composite build-ups were made and cut into dentin-composite sticks. μTBS and failure modes were analyzed. Nonparametric statistical analyses (α = 0.05) were performed for comparison of the five groups within each type of adhesive as well as between the two adhesive systems used. The use of an ER instead of a SE adhesive system resulted in significantly higher μTBS for all irrigation protocols except for group 1 (NaOCl–EDTA–NaOCl) and 2 (NaOCl–NaOCl–EDTA). A statistical difference between the five different endodontic irrigation protocols was only found within the SE adhesive group, where group 1 (NaOCl–EDTA–NaOCl) achieved highest values. The use of an ER adhesive system cancels out the effect of the endodontic irrigation solution. The highest μTBS was achieved when using a NaOCl–EDTA–NaOCl-irrigation protocol in combination with Clearfil SE Bond, which shows that the selection of the endodontic irrigation should match the corresponding SE adhesive system.

## Introduction

A tight coronal restoration is crucial for the prognosis of endodontically treated teeth, as microorganisms along leaky restorations can directly reach the root filling and affect periapical health^1,2^. Since composite materials can be adhesively bonded to the tooth substance, they are often used for the definitive coronal seal of endodontically treated teeth^3^. Composite restorations are bonded to the tooth to replace lost dental hard tissue and prevent marginal leakage^4^. For long-term success, Ray and Trope^5^ even attributed greater importance to the coronal restoration than to the endodontic treatment itself.

In addition to mechanical root canal instrumentation, endodontic irrigation solutions are needed to disinfect infected hard and soft tissue. Endodontic irrigation solutions can remove debris produced during mechanical instrumentation and dissolve tissue residues. Sodium hypochlorite (NaOCl) and ethylenediaminetetraacetic acid (EDTA) are classic representatives of such endodontic irrigation solutions^6^.

NaOCl has antimicrobial effects, proteolytic activity, and debridement properties and is thus a widely used endodontic irrigation solution that dissolves organic substances like necrotic pulp tissue or biofilm^7,8^. For highest efficiency, NaOCl should be used in concentrations above 1–2% and should always be kept in motion^9,10^. The nonspecific proteolytic activity of NaOCl dissolves organic components of dentin and may thus reduce its elastic modulus and flexural strength^11^. However, NaOCl does not affect the inorganic part of dentin^8^.

EDTA serves as chelator and is generally used in concentrations of 15 or 17%. It removes inorganic components inside the root canal system^12^. For sufficient removal of inorganic components, an exposure time of at least 2 min is recommended^6,13^. Mechanical instrumentation and rinsing with NaOCl causes a so-called smear layer, containing ground dentin and predentin, pulpal remnants, and microorganisms^13^. Removal of the smear layer by means of a chelating agent is important because microorganisms and their metabolic products remain in it and may lead to persistent infection^14^. However, EDTA has also been shown to soften dentin due to its demineralizing effects on inorganic components^13^.

In recent years, a new combined all-in-one irrigation solution (Dual Rinse HEDP, Medcem, Weinfelden, Switzerland) has gained increasing interest in endodontic community as it promises novel properties based on its “continuous chelation” concept^15^. Dual Rinse HEDP consists of an oxidation-resistant chelator (1-hydroxyethane 1,1-diphosphonic acid (HEDP)), which is directly diffused in a NaOCl solution^16^. The aim is to simplify endodontic treatment by avoiding the need of multiple irrigation solutions. NaOCl does not lose its antimicrobial effect when HEDP is added^16^. This is an advantage compared to other chelators which compromise the antibacterial activity of NaOCl^17^. Thus, Dual Rinse HEDP combines antimicrobial and proteolytic effects of NaOCl with decalcifying effects of HEDP within a single endodontic irrigation solution^18^.

The chemical effects of endodontic irrigation solutions on root canal dentin described above might also affect coronal dentin, which gets contaminated by these chemicals during endodontic treatment. The aim of this in vitro study was therefore to investigate the influence of different endodontic irrigation solutions and protocols on the micro-tensile bond strength (μTBS) of composite restorations to dentin when using an etch-and-rinse (ER) or self-etch (SE) adhesive system. The null hypothesis claimed that the endodontic irrigation protocol would not affect the μTBS of the adhesives to dentin.

## Materials and methods

### Specimen preparation

Eighty extracted human molars were collected for this study. The teeth had medical indications for the extraction, which was performed during routine dental treatment. Before collection of the teeth for study purposes, patients gave written informed consent about the further use of their teeth as anonymized biological material.

Irreversible anonymization of the teeth was done directly after extraction.

The study was thus performed in accordance with the Federal Act on Research involving Human Beings (Human Research Act; article 2, paragraph 2) and authorization from the ethics committee was waived (BASEC-Nr. Req-2022-01010/2022-01029).

Only teeth free of caries or damages were included in this study. Biological residues such as soft tissue, dental calculus or bone were removed. The teeth were randomly allocated in two main groups, which were further split into five different experimental subgroups each. Until the experiment, the teeth were stored in tap water at 5 °C.

Further handling was simplified by fixing the teeth on custom-made carriers (Wenka, Karl Wenger SA, Courgenay, Switzerland) using light-curable resin (LC Block-Out Resin, Ultradent Products Inc., South Jordan, UT, USA). The fixed teeth were embedded on the carrier with self-curing acrylic resin (Paladur, Heraeus Kulzer, Hanau, Germany) covering two thirds of their roots.

To reach occlusal dentin, the tooth crowns were removed below the level of fissures with a low-speed precision cutter (IsoMet, Buehler, Lake Bluff, IL, USA) and a saw wheel plated with diamonds (M0D10, Struers, Birmensdorf, Switzerland; diameter: 102 mm, thickness: 0.3 mm). The dentin was then ground with 180 grit silicon carbide paper (Buehler-Met II, Buehler, Lake Bluff, IL, USA) to create a roughening effect similar to that of an 80 μm diamond bur^19^. For this purpose, a polishing machine (Planopol-2, Struers, Ballerup, Denmark) was used at low speed (150 rpm) and under constant water cooling. A stereo microscope (Stemi 2000, Carl Zeiss, Feldbach, Switzerland; 15× magnification) was used to verify that enamel was completely removed and pulp was not opened.

### Endodontic irrigation solutions and protocols

**Figure 1:**
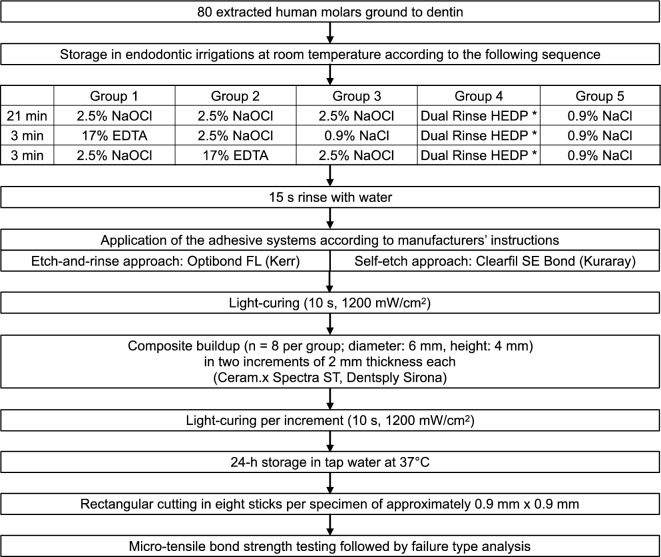
Flowchart of the study. * 2.5% NaOCl + 9% HEDP.

Four endodontic irrigation protocols and a control irrigation (0.9% NaCl) were investigated. The experimental setting with the different endodontic irrigation solutions and protocols is shown in Fig. 1.

Each specimen was stored inside baths with 40 ml of the respective irrigation solution for the time defined per group. In the groups with a change of irrigation solution, the specimens were carefully dried with a paper towel before changing the irrigation solution to avoid cross contamination between the baths. After a total of 27 min in the endodontic irrigation solutions^20^, the specimens were rinsed with water for 15 s. The specimens were stored in tap water at room temperature before composite buildup.

### Composite buildup

As described in section Specimen Preparation, the study included two main groups, which were divided into five subgroups each. In the main groups, two different adhesive systems were used according to the manufacturers instructions. Four endodontic irrigation protocols and a control group (0.9% NaCl) formed the subgroups, which were the same in the two main groups.

A three-step adhesive system (Optibond FL, Kerr, Orange, CA, USA) was applied in the first main group. After etching with phosphoric acid (Ultra-Etch, Ultradent, South Jordan, UT, USA) for 15 s and rinsing with water for 15 s, the primer was applied to the carefully air-dried dentin. After the primer was dried with air stream, the adhesive was applied, and light-cured for 10 s at 1200 mW/cm^2^ with an LED light-curing unit (Bluephase G2, Ivoclar Vivadent, Schaan, Liechtenstein; emission wavelength range: 385–515 nm).

A self-etch adhesive system (Clearfil SE Bond, Kuraray, Osaka, Japan) was applied in the second main group. After primer application for 20 s and drying with mild air stream, the bonding agent was applied and blown with mild air stream before light-curing for 10 s at 1200 mW/cm^2^.

A custom-made holder was used for the composite build-up. The holder fixed a transparent silicone tube with an inner diameter of 6 mm and a height of 4 mm on the dentin surface. Height markings on the silicone tube were followed to place two 2 mm thick composite increments (Ceram.x, Spectra ST, Dentsply Sirona, Konstanz, Germany; shade: A2; lot no.: 2108000620). Light-curing was performed at a distance of 1 mm from each composite increment by using the light-curing unit described above (10 s, 1200 mW/cm^2^). Radiant exitance of the light-curing unit was checked regularly with a dental radiometer (Field-MaxII-TO, Coherent; Santa Clara, CA, USA). After composite buildups, the teeth were dark-stored in tap water at 37 °C for 24 h.

### Micro-tensile bond strength test

The dentin–composite specimens were cut in two directions to obtain rectangular sticks. For this purpose, a water-cooled precision saw (Accutom-50, Struers, Birmensdorf, Switzerland) with a diamond-coated saw blade (M0D10, Struers, Birmensdorf, Switzerland; diameter: 102 mm, thickness: 0.3 mm) was used. The eight most central dentin–composite sticks were marked with a waterproof pen before cutting them off the teeth parallel to the occlusal surface. Complete absence of enamel was verified with a stereo microscope (Stemi 2000, Carl Zeiss, Feldbach, Switzerland). 320 sticks per main group and 640 sticks for the whole study were produced and examined. To prevent the dentin from drying out, the sticks were stored in tap water at room temperature until μTBS was measured.

For the further calculation of the μTBS, the cross-sectional area was determined at the level of the adhesive interface between composite and dentin. For this purpose, the edge lengths were measured with a digital micrometer (406-250-30, Mitutoyo AG, Urdorf, Switzerland), and the cross-sectional area was calculated from the product of both edge lengths. The sticks were prepared for μTBS testing according to Armstrong et al.^21^. The composite and the dentin end of the sticks were glued into sandblasted (110 μm aluminum oxide, 4.5 bar) μTBS jigs (Wenka, Karl Wenger SA, Courgenay, Switzerland) with cyanoacrylate glue (No. 17330050; Renfert GmbH, Hilzingen, Germany). To determine μTBS, the sticks were fixed in a universal testing machine (Zwick Roell Z010, Ulm, Germany). A load cell of 500 N was used to carry out a tensile force test at a speed of 1 mm/min until the sticks failed. The μTBS (MPa) was calculated for each stick by dividing the load at failure (N) by the previously determined bonding area (mm^2^).

### Failure mode analysis

The sticks were subjected to a failure mode analysis. Five different failure modes were distinguished: adhesive failure, cohesive failure in dentin, cohesive failure in composite, mixed failure, and pre-test failure. For examination of the failure modes, a stereo microscope (Stemi 2000, Carl Zeiss, Feldbach, Switzerland) with 15× magnification was used.

### Statistical analysis

The sample size calculation was based on a preliminary study (data not shown) in which mean values in the range of 20–30 MPa with standard deviations of about 10 MPa were obtained. Therefore, the power analysis was performed with the aim of identifying a difference of 20% to 25 MPa. This results in an effect size d of about 0.5 and a power of about 80 % would be achievable with n = 60 per experimental group. Since eight dentin-composite sticks could be obtained from each tooth, eight teeth per experimental group were used to obtain the final sample size of 64 sticks (8 sticks per tooth × 8 teeth). The total number of dentin-composite sticks was 640 (64 sticks per group × 10 groups).

For statistical analysis, µTBS values of zero were assigned to all pre-test failures^21^. Nonparametric statistical analysis was performed since the Shapiro–Wilk test and normal Q–Q plots indicated significant violations of the normality assumption. Comparisons among the five experimental groups within each type of adhesive were performed using the Kruskal–Wallis test with Dunn’s post-hoc procedure and Bonferroni adjustment for multiple comparisons. Pairwise comparisons between the ER and the SE adhesive system within each group were performed using the Wilcoxon signed-rank test. Statistical analysis was performed using SPSS (version 25; IBM, Armonk, NY, USA) at an overall significance level of α = 0.05.

### Ethical approval

The study was conducted in accordance with the Declaration of Helsinki. Ethical review and approval were waived for this study (BASEC-Nr. Req-2022-01010/2022-01029), as it complied with the use of anonymized biological material (Federal Act on Research involving Human Beings (Human Research Act; article 2, paragraph 2)).

### Patient consent statement

Informed consent was obtained from all donors of teeth.

## Results

### Micro-tensile bond strength

**Figure 2:**
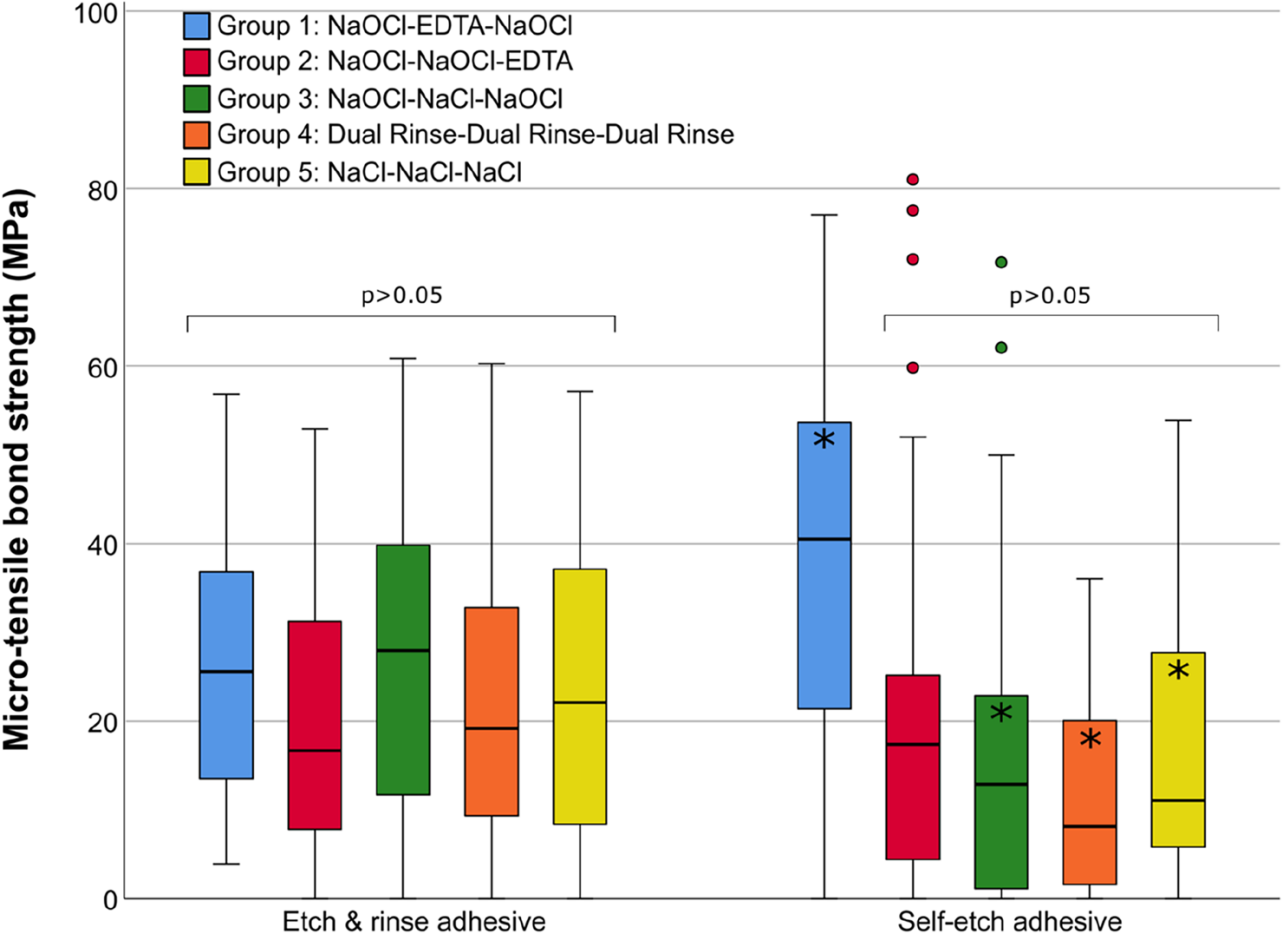
Micro-tensile bond strength (MPa) to dentin after treatment with the different endodontic irrigation protocols using an etch-and-rinse or self-etch adhesive system. Values within square brackets do not differ significantly at the 0.05 level. Asterisks denote statistically significant differences between the etch-and-rinse and self-etch adhesive. The boxplots show the medians (bold black lines) and the boxes represent the 25% and 75% quartiles, while the whiskers represent 1.5 × interquartile range (IQR), or minima and maxima of the distribution if below 1.5 × IQR. Outliers are presented by circles.

Figure 2 shows µTBS to dentin after treatment with the different endodontic irrigation protocols.

For the ER adhesive, all five groups showed statistically similar µTBS values (p = 0.068). For the SE adhesive, group 1 showed significantly higher values (p < 0.001) compared to the remaining four groups, which did not significantly differ among themselves (p = 0.471). Pairwise comparisons between the ER adhesive and the SE adhesive showed significant differences for all groups (p < 0.001) except for group 2 (p = 0.221). For group 1, the SE adhesive achieved significantly higher µTBS than the ER adhesive, while for groups 3–5, µTBS of the SE adhesive was significantly lower compared to the ER adhesive.

### Failure mode analysis

**Figure 3:**
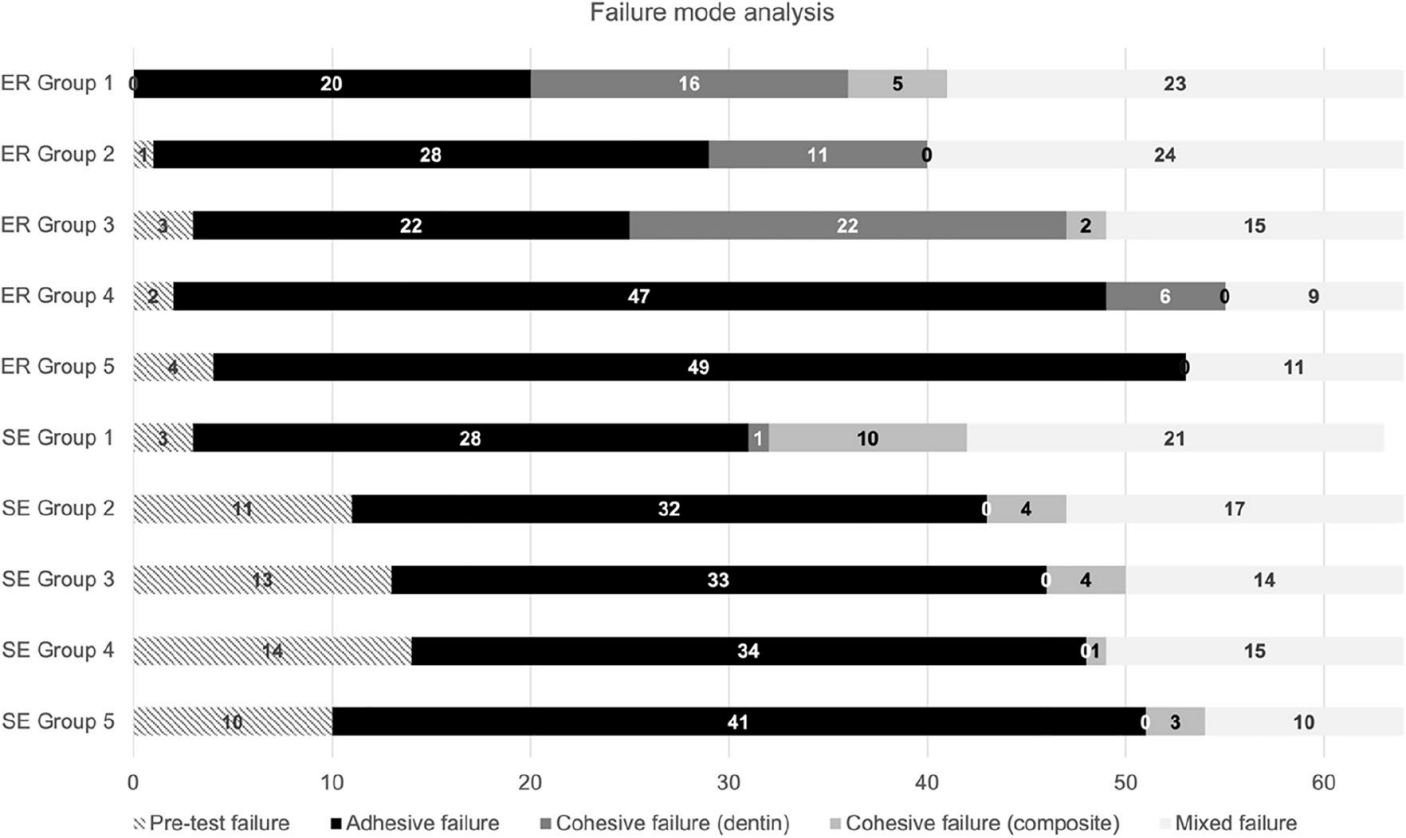
Distribution of failure modes per group, given in total numbers.

The distribution of failure modes per group is given in Fig. 3. Across all groups, the predominant failure mode was adhesive failure. For groups 1–3, the SE adhesive system showed adhesive failures more frequently than the ER adhesive system. It was also shown that in the groups where no NaOCl was used (groups 4, 5), the number of adhesive failures increased for both adhesive systems. Pre-test failures occurred more often when using the SE adhesive system.

## Discussion

Our findings illustrate no significant differences in dentin bond strength between pretreatments with different endodontic irrigation solutions and protocols when using an ER adhesive system. However, when using the SE adhesive system, significantly higher dentin bond strength was observed after pretreatment with the sequence

NaOCl–EDTA–NaOCl than with the other endodontic irrigation solutions and protocols. Thus, the null hypothesis could be partially rejected.

Various endodontic irrigation solutions and protocols exist. Their most important task is to disinfect the non-mechanically manipulated areas of the root canal system^22^. Endodontic irrigation solutions unavoidably also come into contact with the dentin coronal to the root canal system, and affect its properties^8,23^. A tight coronal seal with an adhesive restoration is important to prevent bacteria from re-entering the filled root canal system^3^. Therefore, when selecting an endodontic irrigation protocol, its influence on dentin adhesion should also be considered^24,25^.

The period of 21 min for the main irrigation in the chemo-mechanical preparation of root canals was chosen as clinically realistic according to an earlier study by Marending et al.^20^. According to the same study, the final irrigations were performed for 3 min each. Since both the removal of the smear layer and the decrease in the mechanical properties of the dentin when using EDTA + NaOCl irrigation are time-dependent, different irrigation times have been used in attempts to balance the desirable and adverse effects^26–30^. For example, in vitro studies have used EDTA for 1–5 min^26,27^, 1–10 min^28^, 3 min^29^, and 3–15 min^30^. For the present study, a rinsing time of 3 min was chosen for both EDTA and NaOCl according to the previous study^20^. Within the limitations of the current study, it was not possible to evaluate the efficacy of smear layer removal or possible dentin erosion due to the irrigation protocols used^12,23^. Therefore, it cannot be excluded that the µTBS values obtained were influenced by the impaired mechanical properties of the dentin substrate due to the alternating EDTA and NaOCl irrigation. Nevertheless, there does not appear to have been a significant weakening of the mechanical properties of the dentin, as evidenced by the results of the group with the most aggressive irrigation effect on the dentin (group 1), which did not show a significant reduction in µTBS values compared to the other groups.

The sequence of irrigations and irrigation times were based on the previous study by Marending et al. ^20^. The first irrigation in the sequence, which lasted 21 min, was intended to simulate irrigation during the chemo-mechanical preparation of the root canal. This irrigation was followed by EDTA (3 min) and NaOCl (3 min) in group 1, which was intended as a standard reference protocol for the removal of the smear layer. In group 2, EDTA was used as the final rinse (without a subsequent NaOCl rinse), but a 3 min NaOCl period was added before the final rinse to obtain the same exposure time to NaOCl (24 min) as in group 1. The rinsing protocol for group 3 was created by replacing the 3 min EDTA rinse from group 1 with the NaCl rinse of the same duration. This was intended to exclude the effect of EDTA while maintaining all other parameters as in group 1. Group 4 was only rinsed with Dual Rinse HEDP for the same total time (21 + 3 + 3 min) as all other groups. In group 5, the same total time (21 + 3 + 3 min) was used with a single irrigation (NaCl) as a negative control. It was assumed that the NaCl rinse had no significant effect on the smear layer and the structural integrity of the dentin and did not interact with any of the other irrigations used^26^.

Except for groups 1 (NaOCl–EDTA–NaOCl) and 2 (NaOCl–NaOCl–EDTA), the ER adhesive system resulted in significantly higher dentin bond strength values than the SE adhesive system, which is in accordance with other studies investigating the difference in bond strength between ER and SE adhesive systems^31–33^. Phosphoric acid etching demineralizes the dentin surface up to a few micrometers where it exposes a collagen mesh. With these exposed collagen fibrils, an optimal hybrid layer can be formed by infiltration of hydrophilic monomers of the ER adhesive system^32,34^.

At the same time, no significant differences in dentin bond strength were observed within the ER adhesive system (Optibond FL) regardless of the endodontic irrigation protocol used. This could be explained by the altered dentin surface after phosphoric acid etching^35^, which removes the smear layer and superficial peritubular dentin leading to an opening of dentinal tubules in a funnel shape^8,23,36^. Since endodontic rinsing solutions might have a shallower effect on dentin than phosphoric acid, it can be assumed that the affected layer of dentin gets removed through subsequent phosphoric acid etching.

Within the SE adhesive system (Clearfil SE Bond), significantly higher bond strength was found for group 1 (NaOCl–EDTA–NaOCl) compared to all other groups. Clearfil SE Bond is a mild adhesive system (pH 2.0), which does not lead to excessive decalcification and alters dentin surface less than phosphoric acid^37^. This could explain why differences in dentin bond strength occurred between the different endodontic irrigation protocols when the SE adhesive system was used.

In group 1, the dentin surface is first decollagenized by NaOCl irrigation, which exposes the smear layer. Subsequent irrigation with EDTA finally removes the smear layer and dissolves inorganic components such as calcium^38^. The final NaOCl irrigation cleans and decollagenizes dentin again down to deeper layers and thus exposes dentin tubules^39,40^. This might have exposed calcium precipitates^23^, to which 10-methacryloyloxydecyl dihydrogen phosphate (10-MDP) contained in Clearfil SE Bond could bind, thus leading to superior dentin bond strength with this irrigation protocol.

In group 2, EDTA was the last irrigation affecting dentin. Regarding dentin structure, there is an organic part with collagen fibrils, which is covered by an inorganic part with hydroxyapatite. EDTA removes the inorganic part and exposes collagen fibrils, but it does not interact with the organic part. As EDTA was the last irrigation applied in group 2, the inorganic hydroxyapatite part around the collagen fibrils had not been removed. Thus, the NaOCl used before EDTA application could not interact adequately with the organic part and decollagenize the dentin^23^.

In group 3, NaCl, which has no effect on the dentin surface, was used as a second irrigation instead of EDTA. NaCl does not remove the smear layer^41^ and thus the final NaOCl rinse might not have been able to decollagenize dentin sufficiently^42^. This could be an explanation for the lower dentin bond strength in group 3 compared to group 1 when the SE approach was used.

Group 4 (Dual Rinse HEDP: 2.5% NaOCl + 9% HEDP) showed no significant differences in dentin bond strength compared to groups 2, 3 and 5, where no HEDP was contained. It is likely that the deproteinizing effect of the Dual Rinse HEDP solution was too mild to remove enough of the smear layer^43,44^. Since HEDP is added as a salt, it increases the density as well as the surface tension of the irrigation solution^42^. In the setting of our study, the liquid was stirred, but not actively manipulated with instruments or even ultrasound as in a clinical situation, which might explain the insufficient effect of the added HEDP salt.

The control group (group 5) had no impact on dentin bond strength and showed similar dentin bond strength as groups 2 (NaOCl–NaOCl–EDTA), 3 (NaOCl–NaCl–EDTA) and 4 (Dual Rinse HEDP).

Group 1 (NaOCl–EDTA–NaOCl) was the only group which showed higher dentin bond strength with the SE than with the ER adhesive system. This might be explained by the fact that 10-MDP contained in Clearfil SE Bond was able to bind to dissolved calcium precipitates and form stable calcium-monomer complexes^45^. In an ER approach, however, phosphoric acid etching removes the calcium components completely, which is why no chemical interaction between calcium and functional monomers can occur^46^.

A limitation of the present study is that only one ER and SE adhesive were included, and μTBS was only measured 24 h after placement of the composite restorations. Further studies on the long-term dentin bond strength of various other adhesive systems after endodontic irrigation are thus needed to be able to draw more general recommendations.

## Conclusion

Based on the results of this in vitro study, it can be concluded that the endodontic rinsing irrigations and their sequence affect dentin bond strength when using the SE adhesive, with the sequence NaOCl–EDTA–NaOCl resulting in the highest bond strength values. The tested ER approach resulted in similar dentin bond strength values with the different endodontic irrigation protocols. Through phosphoric acid etching, the influence of the endodontic irrigation protocols on dentin bond strength thus reduces into the non-significant range.

## Data Availability

The datasets generated during and/or analyzed during the current study are available from the corresponding author on reasonable request.
